# Specific detection of *OCT4* isoforms in inflammatory bowel disease

**DOI:** 10.1186/s13099-015-0073-1

**Published:** 2015-10-01

**Authors:** Maria Maragkoudaki, Anna Vaiopoulou, George E. Theodoropoulos, Evangelia Legaki, Leonardo A. Sechi, George Karamanolis, George Zografos, Maria Gazouli

**Affiliations:** First Department of Pediatrics, Athens University Medical School, “Aghia Sophia” Children’s Hospital, Athens, Greece; Laboratory of Biology, Department of Basic Medical Sciences, School of Medicine, University of Athens, Michalakopoulou 176, 11527 Athens, Greece; Colorectal and Inflammatory Bowel Diseases Unit, First Department of Propaedeutic Surgery of Athens Medical School, Athens, Greece; Sezione di Microbiologia e Virologia, Dipartimento di Scienze Biomediche, Università degli Studi di Sassari, Sassari, Italy; Department of Surgery, “Aretaieio” University Hospital, Athens, Greece

**Keywords:** IBD, Crohn’s disease, Ulcerative colitis, Stem cells, Mobilization

## Abstract

**Background:**

Developmentally early cells are mobilized into peripheral blood in Crohn’s disease (CD) patients. OCT4, is considered to be important in sustaining the pluripotency of stem cells. *OCT4* splicing variants are differentially expressed in pluripotent and non-pluripotent cells. Our study aims to investigate the expression pattern of OCT4 variants and SOX-2, an essential factor implicated in self-renewal and pluripotency, in tissue and blood samples from patients with IBD.

**Methods:**

Peripheral blood and tissue samples were collected from patients with active CD and ulcerative colitis (UC), and from healthy individuals. OCT4 expression was documented by Western blot, immunohistochemistry and by reverse transcription-real-time PCR. *OCT4* isoform determination was documented using specific primers. *SOX*-*2* expression levels were also evaluated.

**Results:**

OCT4 protein levels were significantly higher in CD tissue samples than in CD blood samples, and in UC tissue samples. OCT4 protein was localized mainly in the cytosol. In all samples, only the *OCT4* pseudogenes and the *OCT4B1* variant were detected. *OCT4B1* expression levels were elevated in both tissue and blood samples from CD and UC cases compared to healthy controls. In CD patients only *SOX*-*2* mRNA levels were found slightly increased compared to healthy controls.

**Conclusion:**

Our results suggest that OCT4 is expressed in patients with IBD. Furthermore, we found the presence of the *OCT4B1* isoform in IBD in both tissue and blood samples. Our results have shown, that developmentally early cells might be mobilized into peripheral blood as result of tissue damage, indicating a possible role of these cells in repair of injured intestinal tract.

## Background

Crohn’s disease (CD) and ulcerative colitis (UC), known as inflammatory bowel diseases (IBDs), are chronic immuno-related inflammatory diseases of unknown aetiology. However, it has been considered that IBD pathogenesis is the outcome of an aggressive cell-mediated immune reaction to commensal intestinal bacteria at a genetically predisposed host [1]. Mounting evidence has been indicating that IBD pathogenesis may be influenced by the variety of cell types involved such as stem cells (SCs), including defects of their differentiation [2]. SCs contribute to tissue homeostasis, and regeneration after damage [3]. Currently, the idea of SC-based therapies has been considered as a hopeful approach for the treatment of IBD, essentially by the use of hematopoietic SCs (HSCs) and mesenchymal SCs (MSCs) [4]. It has been supported that cells expressing markers for MSCs, HSCs, fibrocytes, enthothelial progenitors were identified in peripheral blood (PB) during tissue/organ damage, inflammation, and pharmacological therapy [5, 6]. Interestingly, Marlicz et al. [7] reported that cells expressing markers for MSCs, EPCs, and Oct-4 + Nanog + SSEA-4 + CXCR4 + lin-CD-45-very small embryonic-like stem cells (VSELs) are mobilized into PB in CD patients, suggesting that these cells might be important in the development and regeneration of gastrointestinal epithelium in IBD [7].

Regarding OCT4, a POU-domain transcription factor is considered to be a critical factor for the pluripotency of human embryonal stem (ES) and induced pluipotency stem (IPS) cells [8]. As cells differentiate and lose pluripotency, *OCT4* is not expressed. The human *OCT4* gene, situated on chromosome 6, consists of five exons and can be alternatively spliced into three basic isoforms *OCT4A*, *OCT4B*, and *OCT4B1*, and produce four proteins OCT4A, OCT4B-190, OCT4B-265, and OCT4B-164. OCT4A, usually referred as OCT4, is distinctively expressed in the nucleus of ESCs and regulates the stemness of pluripotent cells [9]. Nevertheless, the occurrence of transcribed pseudogenes with high homology to the *OCT4A* sequence raised questions about *OCT4* as a pluripotency marker, and could be a possible source of false positive results or could lead to misinterpretation of RT-PCR experiments addressing in general *OCT4* expression [10, 11]. OCT4B is principally localized in the cytoplasm and cannot maintain the self-renewal and pluripotency of ESCs [12]. The role of *OCT4B* is still unclear; however, Li et al. [13] have recently supported that *OCT4B* functioned as a non-coding RNA, modulating *OCT4A* expression in an miRNA-dependent manner [competing endogenous RNA (ceRNA) regulation] at the post-transcription level in tumour cell lines. The majority of the transcribed *OCT4* pseudogenes have high homology to the *OCT4A* sequence only. Even if the protein product of *OCT4B1* is still not identified, it is known that *OCT4B1* is mainly expressed in human ES and EC cells and is down regulated in accordance to their differentiation [14]. Up to now, a small number of studies on *OCT4* expression distinguish the different spliced isoforms and limited information exists on the expression pattern of every isoform in different cell types.

Taken into account the complexity and variety of *OCT4* spliced variants and protein isoforms, the present study aimed to investigate the expression pattern of *OCT4* isoforms in tissue and blood samples from patients with IBD.

## Methods

### Subjects

Fresh frozen tissue and blood samples from consecutive IBD patients and healthy controls were collected at the Colorectal and Inflammatory Bowel Diseases Unit, First Department of Propaedeutic Surgery of Athens Medical School, Athens, Greece. The diagnosis of IBD was based on criteria (clinical, endoscopic, radiological and pathological) [15]. The histological and immunohistochemical evaluations were done by a “blinded” observer, a pathologist who was unware of the study groups. We examined colon biopsies and blood samples from 12 patients (7 females, 5 males, mean age 45.6 ± 14.5 years) with CD, and from 10 patients (6 females and 4 males, mean age 42.5 ± 14.5 years) with UC. The healthy tissue and blood cohort consisted of 15 volunteers (8 females and 7 males, mean age 51.6 ± 13 years), they underwent standard screening colonoscopy examination and they do not have history of inflammatory, autoimmune and cancer diseases. The study was carried out with ethics committee approval, and all patients and healthy individuals gave written consent.

### RNA extraction and cDNA synthesis

Total RNA was extracted from the tissue and blood specimens using the Trizol reagent (Life Technologies, Grand Island, NY, USA) according to the manufacturer’s instructions. Reverse transcription was performed by incubating 1 mg of total RNA for 1 h at 42 °C in the presence of 500 mg/ml of Oligo dT 12–18, 10 mM deoxyribonucleotide triphosphates, 5× first-strand buffer, 0.1 M dithiothreitol, and 200 U/ml MMLV reverse transcriptase (Invitrogen, Carlsbad, CA, USA). Prior to RT-PCR analysis, all of the RNA samples used had been DNase-treated to reduce the risk of DNA contamination. Quantitative Real-Time Reverse Transcription PCR analysis (Real-Time RT-PCR) for *OCT4* gene expression and for *OCT4* isoform identification was performed as previously described [10].

### Western blot analysis

Frozen tissues were thawed and disrupted with a tissue lyser in RIPA buffer. Total proteins were quantified by the Lowry method. The same procedure was followed for the blood samples also. Equal concentration of proteins was loaded into an 8–12 % SDS-polyacrylamide gel and then electrotransferred. The membranes after blocking were incubated for 16–20 h at 4 °C with the equivalent primary antibody. The detection of the immune complexes after incubation with the appropriate peroxidase-conjugated secondary antibody was performed with the SuperSignal West Pico Chemiluminescent substrate (Pierce). Protein expression of the molecules was expressed as relative intensity, normalized to b-actin. The primary antibodies used in the current study were: rabbit anti-OCT4 (1:1000, Proteintech, 11263-1-AP), rabbit anti-SOX2 (1:1000, Cell Signalling Technology, 2748) and rabbit anti-beta actin (1:1000, Rockland antibodies & assays, 600-401-886), and the secondary antibody: anti-rabbit HRP (1:1000, Lifespan Biosciences, LS-C56309).

### Immunohistochemical analysis

Formalin-fixed paraffin-embedded (FFPE) tissue sections were deparaffinized in xylene (Carlo-Erba Reagents, Milano, Italy) and rehydrated in graded alcohol. Endogenous peroxidase was deactivated by incubation with a solution of 3 % H_2_O_2_ (Sigma-Aldrich, St. Louis, MO, USA) in methanol (AppliChem GmbH, Darmstadt, Germany) for 10 min. Six microwave heating cycles (700 W, 4 min/cycle) with 0.01 Μ citrate buffer (pH 6.0) were used for antigen retrieval. Power Block reagent (Biogenex, USA) was used for 10 min to block non-specific protein binding. Sections were subsequently incubated with rabbit anti-OCT4 (1:100, Proteintech, 11263-1-AP) primary antibody. The sections were then incubated with One-Step Polymer HRP Reagent (Biogenex, USA) secondary antibody for 30 min. Sections were developed for 1 min in DAB (Biogenex, USA), counterstained in Gill’s hematoxylin (Sigma-Aldrich), subsequently dehydrated, mounted and studied by light microscopy using a Leica CTR MIC microscope.

### Statistical analysis

The statistical analyses were done using GraphPad v. 3.00 (GraphPad Software, San Diego, CA, USA). Experiments were performed in triplicates. Positivity rates and differences in expression among the groups were calculated using the Chi square test and the non-parametric Wilcoxon rank sum test, respectively. Probability values of <0.05 were considered significant.

## Results

**Fig. 1:**
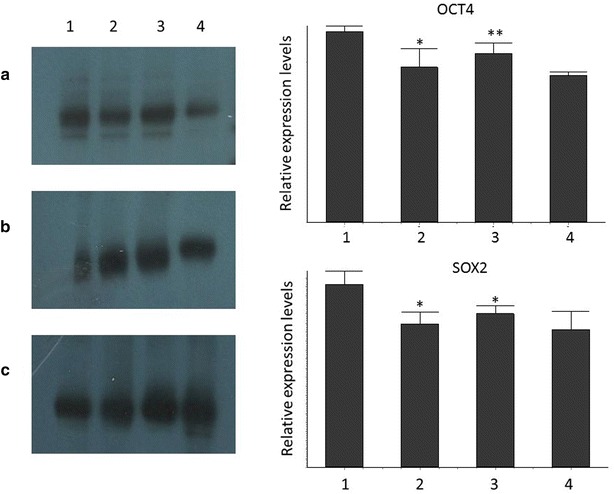
Representative figure of Western blot analysis of OCT4 (**a**), SOX2 (**b**) and b-actin (**c**) proteins, and relative expression of OCT4 and SOX2. Samples *1* CD tissue, *2* CD blood, *3* UC tissue, *4* UC blood. *p < 0.05; **p < 0.01

**Fig. 2:**
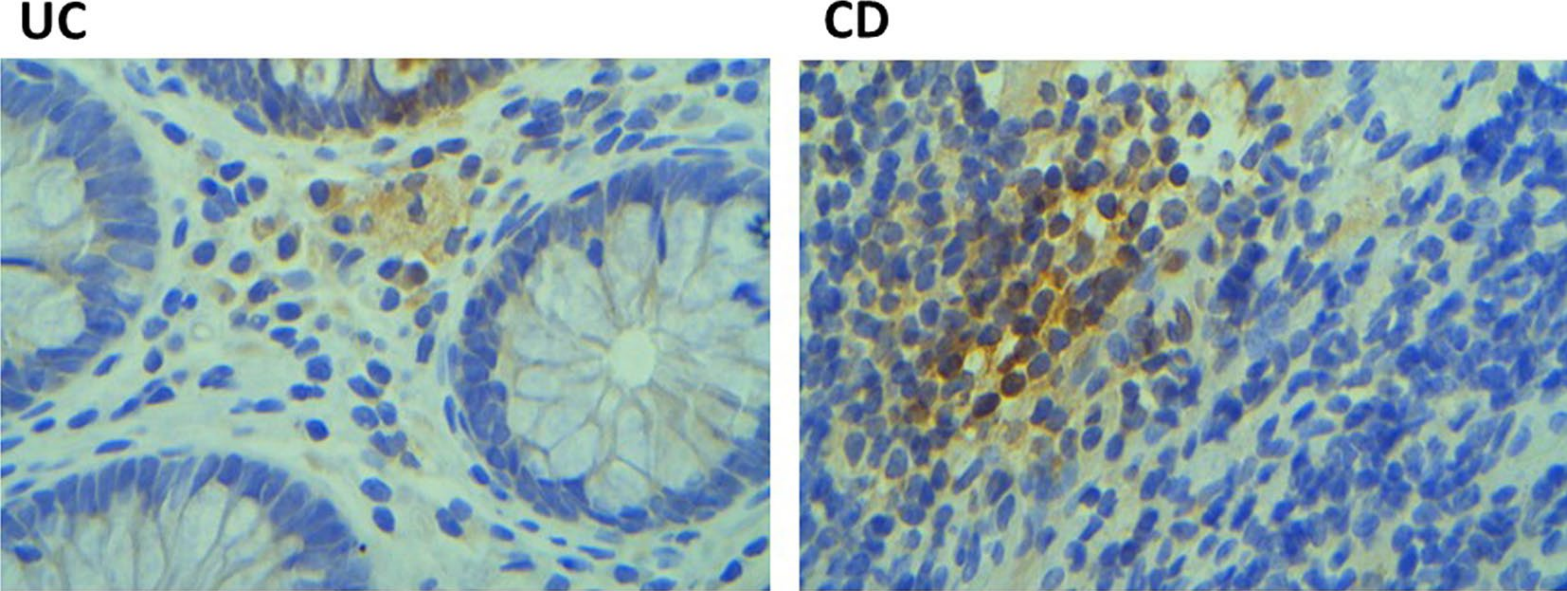
Representative immunohistochemical OCT4 expression analysis of CD and UC tissues

We first analysed the expression of OCT4 protein levels in blood and tissue samples from CD and UC patients. As indicated in Fig. 1, OCT4 was expressed in both tissue and blood samples from CD and UC cases. OCT4 protein levels were significantly higher in CD tissue samples compared to CD blood samples (p < 0.05) and to UC tissue samples (p < 0.01). We also examined the expression of the ESC marker SOX-2 with the aim of determine if the OCT4 expression pattern related to a stem cell phenotype also. Similarly SOX-2 was significantly higher in CD tissue samples compared to CD blood samples (p < 0.05) and to UC tissue samples (p < 0.05). Interestingly, as indicated in Fig. 2 OCT4 protein was expressed mainly in the cytosol in both CD and UC cases, which is different from the description about OCT4 as a nuclear protein. It is known that the different *OCT4* isoforms can be distinguished by their different subcellular localization.

**Fig. 3:**
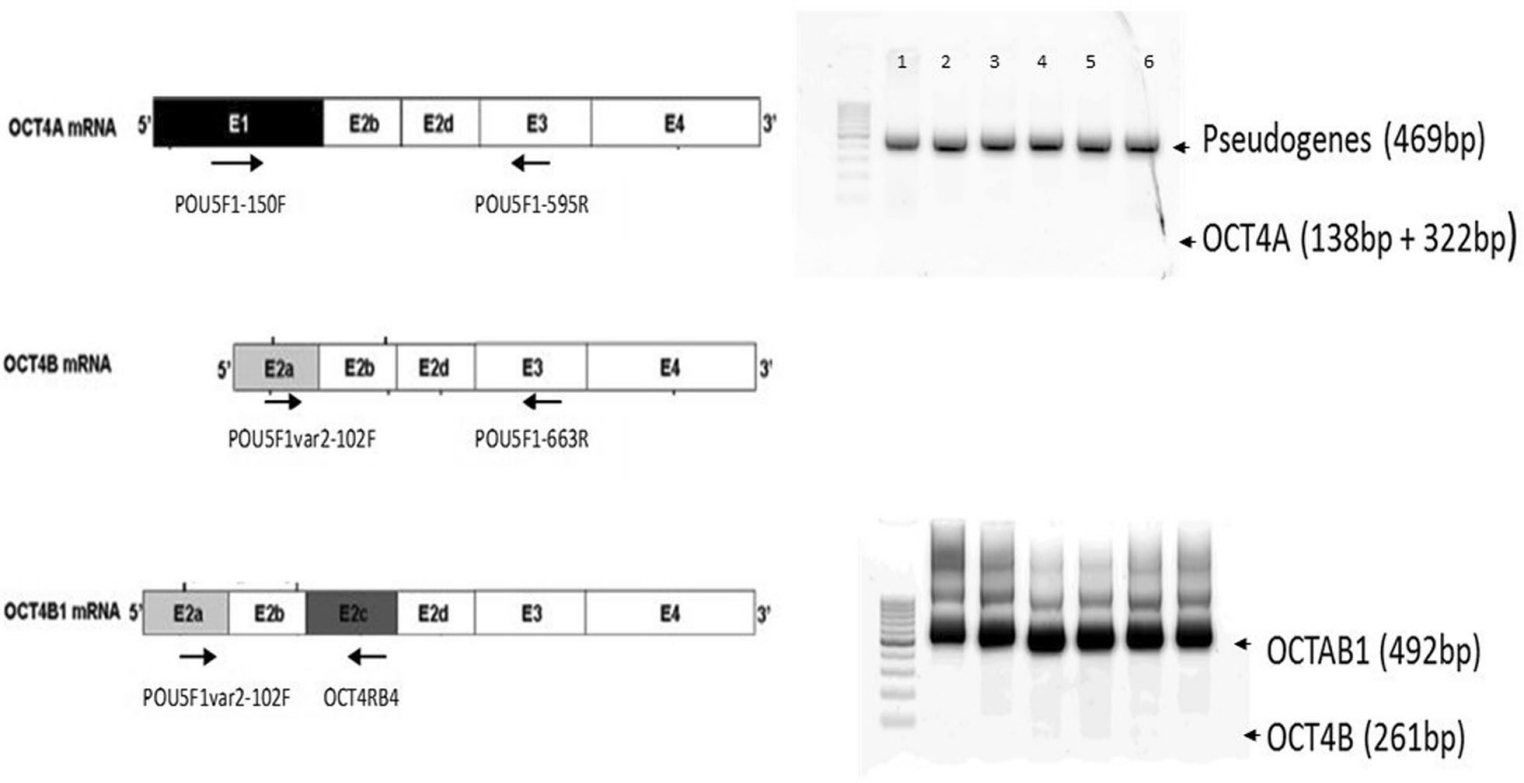
Schematic representation of representative quantitative real-time reverse transcription PCR analysis for OCT4 isoform identification. The primers and the methodology used was performed as previously described [10, 14, 16]. Samples *1*, *2*, *3* were from tissues of healthy, CD and UC, respectively. Samples *4*, *5*, and *6* were from blood of healthy, CD and UC, respectively

**Fig. 4:**
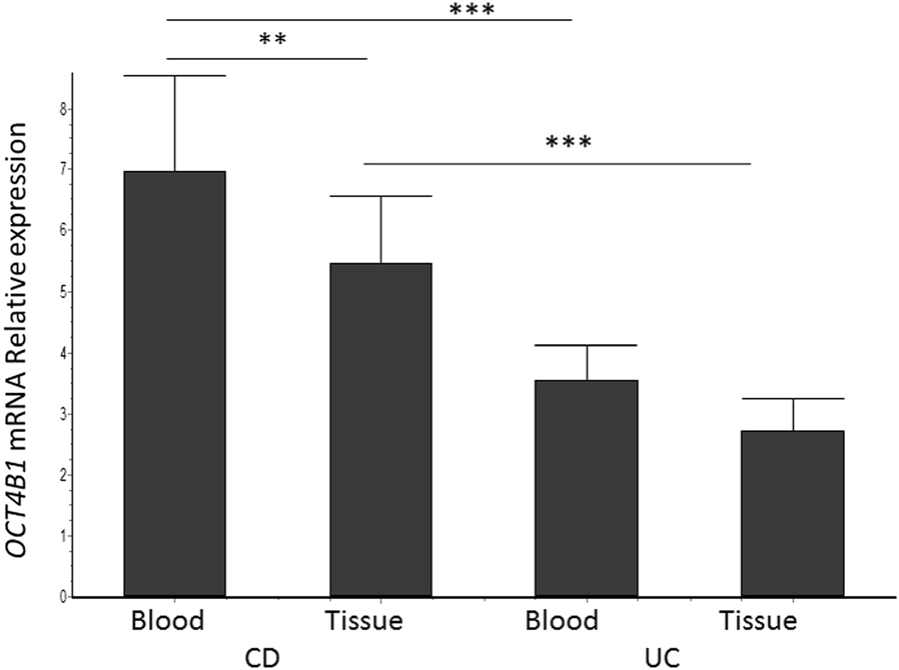
Fold increase of *OCT4B1* mRNA from CD, UC compared to healthy tissue and blood samples. Relative expression level was measured by quantitative real-time RT-PCR

In order to reliably discriminate isoforms *OCT4A* from *OCT4B*, *OCT4B1* and all the known pseudogenes we used the RT-PCR method followed by restriction fragment analysis and *OCT4B1* specific primers as previously described [10, 14, 16]. As indicated in Fig. 3 in all cases only the *OCT4* pseudogenes and the *OCT4B1* variant were detected. The expression pattern of *OCT4B1* variant in CD, UC and healthy tissues and blood samples was further examined. As indicated in Fig. 4, *OCT4B1* expression levels were elevated in both tissue and blood samples from CD and UC cases compared to healthy controls. Particularly, in blood samples *OCT4B1* was expressed 6.95 ± 1.59-fold greater in CD and 3.55 ± 0.57-fold greater in UC compared with healthy controls. Similar results were obtained in tissues samples, also (5.45 ± 1.12-fold greater in CD and 2.74 ± 0.53-fold greater in UC, respectively). The *OCT4B1* mRNA levels were higher in blood samples compared to tissue samples from CD patients. The samples from CD patients expressed higher levels of *OCT4B1* mRNA compared to respective samples from the UC patients. The mRNA levels of *SOX*-*2* were found slightly increased compared to healthy controls, in both blood (0.91 ± 0.17-fold) and tissue samples (0.84 ± 0.14-fold) of CD patients only.

## Discussion

In our study, Western blot analysis of CD and UC tissues and blood samples showed the expression of OCT4 and SOX-2. Thus, in agreement with recent data from Marlicz et al., our findings indicate that stem-like cells might exist in the tissue and blood samples from CD and UC patients [7]. Even if, the expression of OCT4 has been reported mostly in human cancers and cancer cell lines, its expression in IBD has not been systematically investigated. In the present study, IHC analysis of CD and UC tissues showed that positive OCT4 staining was mainly found in the cytoplasm, which is different from the description that OCT4 is a nuclear protein. Since it is known that *OCT4* isoforms can be distinguished by their specific subcellular localization [17], in order to characterize *OCT4* isoform and all its presently known pseudogenes, we used RT-PCR/restriction digestion analysis, as previously described [10, 16]. Our findings suggest that *OCT4A* and *OCT4B* isoforms are not expressed in our IBD cases however the presence of the *OCT4* pseudogenes was found instead. It has been suggested that the transcription of pseudogenes could regulate *OCT4* gene activity [18]. It is interesting to notice that the novel *OCT4* variant nominated as *OCT4B1* was universally expressed in IBD. Atlasi et al. [14] suggested that while ESCs/ECs are expressed at very low levels the novel *OCT4B* isoform, i.e. the *OCT4B1* variant is greatly expressed in these cells [7]. Consequently, our findings support a possible association between *OCT4B1* expression and the presence of a pluripotent/undifferentiated state of stem cell-like cells in IBD cases, and support that the expression of *OCT4* observed in the IBD cases is due to the expression of the *OCT4B1* isoform. *OCT4B1* endows an anti-apoptotic property, especially under stress conditions [19–21]. The inhibition of apoptosis in an important regulatory pathway implicated in the gut immune system and IBD pathogenesis [22]. However, the mechanism by which *OCT4B1* regulates the apoptotic pathway is not yet clarified. The mRNA levels of the *OCT4B1* were found to be higher in blood samples compared to tissue samples of both CD and UC cases.

It is known that stem cells are mainly derived from bone marrow, and are circulating continuously at a low level in peripheral blood. Hematopoietic and non-hematopoietic stem cells can be mobilized into peripheral blood. These cells are mobilized and circulate during tissue/organ injuries. It has been suggested that mobilized cells have a potential role in brain regeneration after stroke, skin burn injury, and acute myocardial infarction [23]. Mobilized stem cells have multiple functions. They contribute to tissue regeneration and to inflammation control by secreting several cytokines that suppress the local immune response [24–26]. In intestine, tissue regeneration may be contributed by stem cells residing in intestinal crypts. Moreover, the immune response suppressing role of these cells makes them a potential therapeutic target for inflammatory diseases such as IBD which is a disease with abnormal mucosal immune response. However, various causes may influence the number of circulating stem cells, such as local inflammation, strenuous exercise, tissue damage and pharmacological factors [6]. It is lately reported that immunosuppressants such as azathioprine, affect stem cell migratory behaviour and influence their therapeutic ability [27].

## Conclusion

In conclusion, our findings show that the presence of stem cells in peripheral blood is expected. Furthermore, we found the presence of the *OCT4B1* isoform in IBD in both tissue and blood samples. Our results have shown that patients with tissue/organ injury and inflammation are likely to modulate a big number of stem cells form bone marrow, which probably either move in the intestine and contribute to regeneration or remain in circulation and regulate immune response.
